# Clinical and Imaging Characteristics of Non-Gestational Ovarian Choriocarcinoma: A Case Report

**DOI:** 10.2174/0115734056386021250520043409

**Published:** 2025-05-26

**Authors:** Xiaofeng Fu, Wei Chen, Jiang Zhu

**Affiliations:** 1 Department of Ultrasound, Women’s Hospital, Zhejiang University School of Medicine, Hangzhou, China; 2 Zhejiang Provincial Clinical Research Center for Obstetrics and Gynecology, Hangzhou, China

**Keywords:** Non-gestational ovarian choriocarcinoma, Imaging characteristics, Human chorionic gonadotropin, Diagnosis, Ovarian germ cell tumor, Vaginal bleeding, Serum human chorionic gonadotropin

## Abstract

**Background:**

Non-gestational Ovarian Choriocarcinoma (NGOC) is an extremely rare and highly malignant ovarian germ cell tumor with nonspecific clinical manifestations, making early diagnosis challenging. At present, detailed reports on the clinical and imaging characteristics of NGOC are scarce. This case report discusses a rare instance of NGOC in a prepubertal adolescent, complemented by a literature review to enhance clinicians’ understanding of its presentation, diagnosis, and treatment.

**Case Presentation:**

A 10-year-old female with no history of menstruation or sexual activity presented with persistent lower abdominal pain and vaginal bleeding. Preoperative imaging revealed a large pelvic mass with heterogeneous echogenicity and vascularity. Serum Human Chorionic Gonadotropin (**hCG**) levels were markedly elevated (>297,000 IU/L).

**Preoperative Imaging:**

Ultrasonography and CT demonstrated a large, heterogeneous, hypervascular adnexal mass with features of necrosis and cystic changes, suggesting malignancy.

**Surgical and Pathological Findings:**

The mass, originating from the right adnexa, was removed *via* laparotomy. Histopathology confirmed NGOC, supported by immunohistochemistry, showing strong positivity for markers like CD146, CK18, HCG, and HPL, along with a high Ki-67 index (>90%).

**Conclusion:**

In young females with no sexual life, significantly elevated HCG levels and imaging findings of a large heterogeneous adnexal mass should raise suspicion for NGOC. Early recognition and multimodal diagnostic approaches, including imaging, biochemical, and pathological assessments, are essential for timely intervention, reducing metastatic risk and improving prognosis. This report contributes to the understanding of NGOC and emphasizes the importance of accurate diagnosis for better patient outcomes.

## 1 INTRODUCTION

NGOC is an extremely rare and highly aggressive germ cell tumor of the ovary, characterized by its nonspecific clinical manifestations that pose significant challenges for early diagnosis. Due to the lack of distinctive symptoms and the rarity of the condition, NGOC is often diagnosed only at advanced stages, which complicates treatment and leads to a poor prognosis. Furthermore, detailed reports on the clinical and imaging features of NGOC are limited, hindering timely diagnosis and optimal management. This case report presents a rare instance of NGOC, supplemented by a review of relevant literature, to provide a comprehensive analysis of its clinical presentation and imaging characteristics. By sharing this case, we aim to enhance the understanding of NGOC among clinicians and imaging specialists, emphasizing the importance of early detection and improving diagnostic accuracy. This report serves as a critical reference to inform future clinical practice and research, contributing to better patient outcomes through earlier recognition and intervention.

## 2 CASE PRESENTATION

### 2.1 General Information

A 10-year-old female patient, with no menstruation or history of sexual life, was admitted with complaints of lower abdominal pain for over three weeks and vaginal bleeding lasting more than two weeks. Three weeks prior to admission, she experienced intermittent, dull, tolerable pain in the right lower abdomen, accompanied by occasional diarrhea but no nausea, vomiting, dizziness, or fever. Two weeks before admission, she developed persistent vaginal bleeding with fresh red blood, though no accompanying abdominal bloating, pain, or fever was reported. One week prior to admission, an abdominal ultrasound at a local hospital revealed a heterogeneous mass in the pelvic cavity, 13.5 × 10.8 × 9.6 cm in size, with clear borders and irregular anechoic areas, raising concern for an ovarian cyst. Her serum hCG level exceeded 297,475 IU/L, prompting a referral to our hospital. Four days before admission, the patient developed a fever (38.6°C), which subsided with antipyretic treatment. Upon examination, her vital signs were stable, and no palpable lymphadenopathy or significant tenderness was observed on abdominal examination. Laboratory test results showed hCG levels of 282,995 IU/L, elevated C-reactive protein (54.2 mg/L), hemoglobin of 93 g/L (suggestive of anemia), and a slightly elevated total thyroxine (192.72 nmol/L).

### 2.2 Preoperative Imaging

#### 2.2.1 Abdominal Ultrasonography

Transabdominal ultrasound revealed a large heterogeneous solid mass in the pelvic cavity measuring 11.7 × 10.5 × 9.7 cm. The mass demonstrated mixed echogenicity, with multiple irregular hypoechoic regions suggestive of cystic or necrotic areas and areas of heterogeneous solid composition. Color Doppler Flow Imaging (CDFI) detected abundant blood flow signals both within and around the mass, indicating significant vascularity. Spectral Doppler analysis further revealed a Peak Systolic Velocity (PSV) of 22.81 cm/s and a resistance index (RI) of 0.28, consistent with the hypervascular nature of the lesion, potentially indicative of malignancy, as shown in Fig. (**1**). These findings, in conjunction with the clinical presentation of persistent vaginal bleeding and markedly elevated HCG levels, strongly suggested a malignant ovarian tumor.

#### 2.2.2 CT Scan

Contrast-enhanced CT revealed a large, roundish solid mass in the pelvic cavity measuring 11.2 × 11.5 × 6.7 cm. The mass demonstrated a heterogeneous internal structure, with a centrally located necrotic area and cauliflower-like solid tissue along its periphery. The peripheral solid tissue exhibited marked enhancement on post-contrast images, as shown in Fig. (**2**), suggesting hypervascularity, a feature commonly associated with malignant ovarian germ cell tumors. Additionally, non-contrast chest CT imaging revealed multiple scattered nodular opacities with relatively well-defined margins in the right lung parenchyma, radiologically suggestive of metastatic lesions. Concurrent non-contrast cranial CT demonstrated no evidence of intracranial abnormalities.

#### 2.2.3 Surgical and Pathological Findings

The patient underwent an exploratory laparotomy that included the removal of the right adnexa, extensive omentectomy, and retroperitoneal lymph node sampling. Intraoperatively, a 12 cm solid mass was identified, with multiple dark protrusions on its surface, which was rough and medium in texture, originating from the right adnexa (the structure of the right ovary and fallopian tube was not clear), with dense adhesions to the posterior uterine wall, uterine fundus, and the left broad ligament. Gross examination revealed that the solid components of the tumor exhibited a dark red color, with some areas of alternating dark red and gray. No fluid discharge was observed. The pathological diagnosis was NGOC of the right ovary, as shown in Fig. (**3**). Immunohistochemical (IHC) analysis supported the diagnosis, showing strong positivity for CD146, CK18, HCG, and HPL, which are markers of choriocarcinoma. The tumor also exhibited a high Ki-67 proliferation index (>90%), indicating aggressive biological behavior. Negative markers included P63, SMA, and EMA, ruling out other differential diagnoses, such as squamous cell carcinoma or mesenchymal tumors.

## 3 DISCUSSION

### 3.1 Ovarian Choriocarcinoma Overview

Ovarian choriocarcinoma is a highly malignant trophoblastic tumor, accounting for approximately 2% of malignant ovarian germ cell tumors [1]. It may originate from (1) primary gestational choriocarcinoma resulting from ovarian pregnancy, (2) metastatic gestational choriocarcinoma originating from other reproductive sites (commonly the uterus), or (3) primary germ cell tumors differentiating into trophoblastic structures. Ovarian choriocarcinoma is, therefore, categorized into two subtypes: gestational choriocarcinoma and NGOC. Gestational choriocarcinoma arises from the malignant transformation of trophoblastic cells and is often secondary to hydatidiform mole, spontaneous abortion, premature delivery, ectopic pregnancy, or normal childbirth. NGOC, on the other hand, is an extremely rare germ cell tumor with trophoblastic differentiation. It is characterized by high malignancy, poor prognosis, and an incidence of less than 0.6% among ovarian malignant germ cell tumors [2, 3].

### 3.2 Clinical Presentation

NGOC predominantly affects children and adolescents and has been reported in both males and females. Its clinical manifestations often include abdominal pain, adnexal masses, irregular vaginal bleeding, and elevated serum HCG levels, closely resembling ectopic pregnancy. Some patients may present with secondary hormonal changes; for instance, prepubertal individuals may exhibit female precocious pseudopuberty [4, 5], whereas others may develop hyperthyroidism, as observed in this case. Notably, markedly elevated HCG levels are a key diagnostic feature of NGOC, often reaching 3–100 times the levels observed in normal pregnancy [6]. By contrast, ectopic pregnancies rarely result in HCG levels exceeding 50,000 IU/L, with an incidence of less than 0.1% for such elevations [7]. Furthermore, serum tumor markers in NGOC are generally within normal ranges, which is consistent with the findings in this case [8].

### 3.3 Imaging Characteristics

NGOC exhibits non-specific ultrasonographic and CT features, typically appearing as a large unilateral solid pelvic mass measuring 8–30 cm in diameter. Some tumor borders are well-defined, while others are poorly demarcated due to adhesions to surrounding tissues. Due to the tumor’s propensity for widespread necrosis, hemorrhage, and vascular invasion, ultrasound often reveals heterogeneous internal echogenicity with scattered irregular cystic areas. CDFI frequently shows rich blood flow signals and low-resistance arterial waveforms, reflecting the tumor’s aggressive vascularity [9]. On contrast-enhanced CT, the mass is often heterogeneous, characterized by marked enhancement of the peripheral solid component but no enhancement of the central necrotic area.

Furthermore, given the intrinsic invasive capacity of trophoblastic cells, characterized by their propensity for vascular wall penetration and destruction, NGOC frequently invades adjacent organs and demonstrates widespread metastasis to distant anatomical sites, with cerebral and pulmonary metastases being particularly prevalent [6]. As in this patient, lung metastases were identified. CT imaging is instrumental in delineating disease extent and identifying potential metastatic spread to distant anatomical sites [2].

### 3.4 Diagnostic Challenges

The preoperative diagnosis of NGOC remains extremely challenging due to its rarity and non-specific clinical and imaging features. Early diagnosis and appropriate initial treatment are critical determinants of prognosis. Recognizing the clinical and ultrasonographic characteristics of NGOC is essential for identifying or suspecting this tumor. When ultrasonography reveals a large pelvic mass with the aforementioned features, serum hCG levels and tumor markers, such as CA125 and alpha-fetoprotein (AFP), should be assessed. Elevated levels of other tumor markers may suggest the possibility of a malignant mixed germ cell tumor. If NGOC is suspected, advanced imaging modalities, such as MRI, CT, and FDG-PET, are necessary for further evaluation.

Gestational and non-gestational ovarian choriocarcinomas differ significantly in their clinical behavior, chemosensitivity, and prognosis, making accurate diagnosis crucial for selecting the optimal surgical and chemotherapeutic approach. A clinical history of pregnancy, amenorrhea, or antecedent gestational trophoblastic disease helps to establish the gestational or non-gestational nature of choriocarcinoma [10]. In females with no sexual history, diagnosis relies on pathological examination and immunohistochemical analysis. For reproductive-age women, DNA polymorphism analysis can distinguish NGOC from gestational choriocarcinoma, as NGOC lacks paternal genetic material [11, 12]. The patient in this report was an adolescent with no history of sexual life. The diagnosis was confirmed postoperatively through histopathological examination and immunohistochemical analysis. Differential diagnosis with other germ cell tumor components primarily relies on surgical pathology, particularly for distinguishing NGOC from yolk sac tumors and embryonal carcinomas.

Regarding future research directions, the development of a comprehensive immunohistochemical marker panel or further investigation into the roles of genomic hybridization and next-generation sequencing in choriocarcinoma may yield clinically valuable molecular markers, thereby improving diagnostic accuracy for NGOC [10, 13]. Additionally, systematic studies analyzing long-term prognosis and survival rates, including the impact of postoperative adjuvant chemotherapy or radiotherapy on patient outcomes, would contribute to the optimization of treatment strategies for this condition.

## CONCLUSION

This case underscores the clinical and ultrasonographic characteristics of NGOC, emphasizing its diagnostic challenges and the importance of combined imaging and biochemical assessments. In young females with no menstruation or sexual life, a marked and persistently elevated hCG level, along with ultrasonographic findings of a large unilateral solid adnexal mass with heterogeneous echogenicity, scattered cystic areas, and rich blood flow signals, should raise suspicion for NGOC. Moreover, early recognition and accurate diagnosis of NGOC are critical for initiating timely treatment, which can significantly reduce the risk of metastasis and improve patient outcomes.

## Figures and Tables

**Fig. (1):**
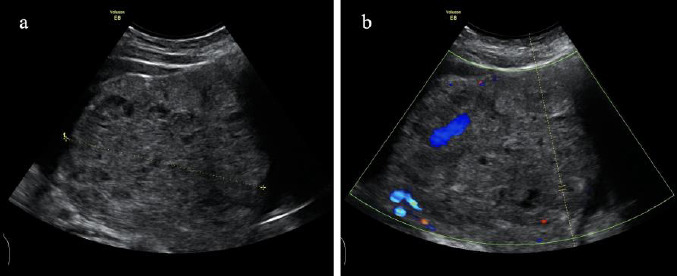
Transabdominal ultrasound image description. (**a**) Two-dimensional ultrasound revealed a large heterogeneous solid mass in the pelvic cavity. (**b**) CDFI showed blood flow signals both within and around the mass.

**Fig. (2):**
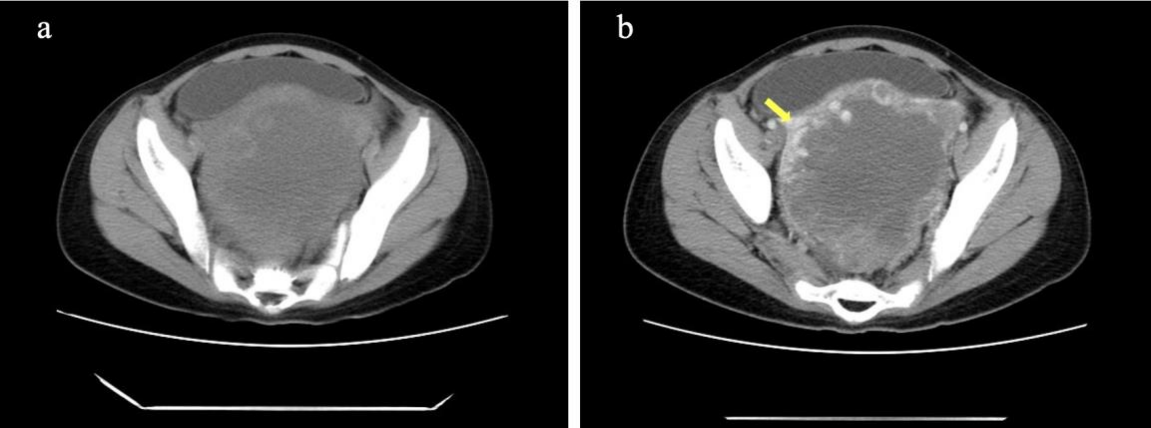
Non-contrast and contrast-enhanced CT image description. Axial non-contrast (**a**) and contrast-enhanced (**b**) CT images at the abdominal level demonstrating a heterogeneously enhancing mass with pronounced enhancement of the peripheral solid component (yellow arrow) and absence of enhancement in the central necrotic area.

**Fig. (3):**
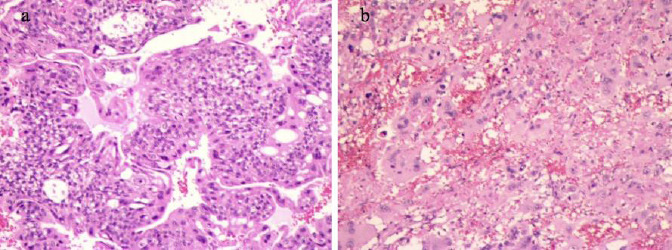
Pathological result description. (**a**) Multinucleated syncytiotrophoblast-like cells with prominent nuclear pleomorphism and hyperchromasia. (**b**) Areas of extensive hemorrhage and necrosis.

## Data Availability

The data and supportive information are available within the article.

## LIST OF ABBREVIATIONS

PSV: Peak Systolic Velocity
CDFI: Color Doppler Flow Imaging
NGOC: Non-gestational ovarian choriocarcinoma
hCG: human chorionic gonadotropin

## STANDARDS OF REPORTING

CARE guidelines have been followed.
